# Impact of economic policy uncertainty, energy intensity, technological innovation and R&D on CO_2_ emissions: evidence from a panel of 18 developed economies

**DOI:** 10.1007/s11356-022-21729-2

**Published:** 2022-07-09

**Authors:** Prince Asare Vitenu-Sackey, Theophilus Acheampong

**Affiliations:** 1 Department of Economics, Strathclyde Business School, University of Strathclyde, Glasgow, G40 QU2 UK; 2 Department of Economics & Aberdeen Centre for Research in Energy Economics and Finance (ACREEF), University of Aberdeen Business School, Aberdeen, AB24 3QY UK; 3 Centre for Energy, Petroleum and Mineral Law and Policy (CEPMLP), University of Dundee, Dundee, DD1 4HN UK

**Keywords:** Technological innovation, Economic policy uncertainty, Carbon emissions, Energy intensity, Environmental Kuznets curve, Pollution halo effect

## Abstract

This study examines the impact of economic policy uncertainty (EPU) and ecological innovation on carbon (CO_2_) emissions in a panel of 18 developed countries from 2005 to 2018 using second-generation time-series panel data techniques. We use three robust long-run estimators, namely two-stage least squares (2SLS), panel generalised method of moments (GMM) and generalised least squares (GLS), to resolve heterogeneity, endogeneity and simultaneity in the panels. We further performed causality tests to ascertain the direction of causality between the variables. Our estimations suggest three innovative findings. First, economic growth contributes significantly and positively to CO_2_ emissions; however, this happens at an optimal level of growth after which carbon emission reduces, indicating that our sample exhibits an inverted U-shaped environmental Kuznets curve (EKC) relationship. Second, the impact of EPU on CO_2_ emissions is diverse: high levels of EPU have a significant influence on CO_2_ emissions only in high-polluting countries but not in low-polluting ones. Thirdly, research and development (R&D), foreign direct investment (FDI), urbanisation and renewable energy (RE) usage were also found to have varying effects on CO_2_ emissions. These findings highlight the heterogeneous relationship between carbon emissions and economic indicators even in advanced economies, as the pollution haven hypothesis (PHH) holds true in high-pollution countries while the pollution halo effect holds for low-pollution ones. A key policy implication of this work is that the quest to mitigate emissions should not be a one-size-fits-all approach because not every country’s urbanisation rate, FDI inflows, R&D and renewable energy consumption directly affect CO_2_ emissions in the face of economic policy uncertainties.

## Introduction

Anthropogenic climate change is having catastrophic negative impacts on public welfare and health outcomes (WHO 2018; Atasoy 2017; Hayes et al. 2018). Since the 1880s, global average surface temperatures have increased by 2 degree Fahrenheit (1 degree Celsius) due to an increase in greenhouse gas emissions (GHGs) (NASA 2020; Lindsey and Dahlman 2021). Some estimates indicate that the last decade alone has seen global average temperatures rise more than 1℃ above pre-industrial levels (Hawkins et al. 2017). These rising surface temperatures have resulted in extreme weather conditions such as droughts, heavy rainfalls, floods and heatwaves — causing severe havoc to ecosystems and humanity (Diffenbaugh 2020). According to NASA (2020), 75% of all GHGs are carbon dioxide (CO_2_) emissions, significantly contributing to global warming.

Thus, carbon dioxide emission abatement is essential to reducing global warming (Jiang et al. 2019). As such, much attention (time, effort and money) in recent times has been dedicated across the globe to finding promising international collaborations and solutions to mitigate global warming. One of these outcomes was the landmark 2015 Paris Climate Agreement Accord. Specifically, the Accord seeks to curb global warming by limiting global warming to well below two (2) — preferably to 1.5 degrees Celsius —compared to pre-industrial levels (UNFCCC 2015). Nevertheless, despite numerous measures to mitigate global warming by championing energy-efficient practices and systems, global CO_2_ emissions are rising (Khan et al. 2020). While CO_2_ emissions dropped by 5.4% in 2020 during the coronavirus (COVID-19) pandemic due to restrictions on movements, emissions are forecast to rise into the foreseeable future - that is, the deepening globalisation, increasing foreign direct investment (FDI) and the resultant increase in energy use in many hitherto energy-poor regions (Tollefson 2021).

Based on this, many environmentalists, policymakers and researchers have attempted to find out the contributing factors to carbon emissions and how to reduce them considering geographical disparities, especially between low-income, middle-income and high-income countries. Broadly speaking, low-income countries and regions typically tend to have relatively less environmental protection. As a result, the pollution haven hypothesis indicates that FDI inflows into these places could negatively affect environmental sustainability. On the other hand, the pollution halo hypothesis posits that FDI inflows can contribute to enhancing environmental sustainability, often in economies or regions with high levels of development. Additionally, wealth affects environmental sustainability: emissions rise at lower wealth levels, peak in the middle and decrease at higher levels. Grossman and Krueger (1995) coined the term “environmental Kuznets curve” (EKC) to refer to this concept.

Since the turn of the century, economic policy uncertainty (EPU), defined as ambiguity and or vagueness in economic policies, has increased. Recent events, including the 2008–09 financial crisis, Brexit, the US-China trade war and the coronavirus (COVID-19) epidemic, have increased global EPU. In its country assessments, the International Monetary Fund (IMF) is reported to have highlighted EPU as a significant driver of poor economic growth (Anser et al. 2021a, 2021b). Multiple studies also demonstrate the economic effects of EPU, namely on the stock market and investment activities (Baker et al. 2016; Sahinoz and Erdogan 2018; Kang and Ratti 2013; Rehman and Apergis 2019; Kang et al. 2014). This conclusion can be drawn since the EPU has a considerable impact on economic output, as captured in various indices. Furthermore, EPU can influence environmental outcomes in addition to economic ones. For example, environmental quality can improve as the economy slows and energy use decreases. EPU-led constraints can also stifle renewable energy, research and development and innovation. In essence, EPU can increase or mitigate ecological threats.

Recently, one study examined the relationship between EPU and environmental quality and presented two possible explanations: (1) influence on consumption and (2) effect on investment (Wang et al. 2020c). According to its consumption effect, economic policy uncertainty minimises the use of energy- and pollution-intensive products. As a result, environmental damage will be mitigated. On the other hand, economic policy uncertainty has a deterrent effect on investment in renewable energy and research and development, resulting in environmental degradation. A small number of academic institutes have examined the effect of economic policy uncertainty on environmental degradation. According to one school of thought, economic policy uncertainty exacerbates environmental degradation (Wang et al. 2020a; Jiang et al. 2019; Adams et al. 2020; Anser et al. 2021a, 2021b), whereas another school of thought holds that economic policy uncertainty mitigates environmental degradation (Adedoyin and Zakari 2020; Syed and Bouri 2021; Chen et al. 2021). Additionally, another study found that economic policy uncertainty has little effect on the ecology (Wang et al. 2020b). Due to the inconsistent findings of some of these earlier studies, additional investigation into the environmental effects of economic policy uncertainty is necessary. This motivates our work.

Furthermore, some authors have studied the relationships between ecological innovation and energy consumption on carbon emissions (Chen and Mkumbo 2020; Fethi and Rahuma 2019; Khan et al. 2020; Mensah et al. 2018; Zhang et al. 2017), and economic policy uncertainty (EPU) and carbon emissions (Adams et al. 2020; Jiang et al. 2019). However, the evidence regarding the subject matter is also inconsistent. Additionally, no recent study or previous study to the best of our knowledge has focused on the impact of technological innovation using the instruments of patent registrations and EPU on carbon emission - in a single study - with a primary motive to assess the overall effect of the levels of innovation level. Here again, this study plugs this identified gap. In this regard, we assess the impact of energy intensity, research and development, technological innovation and EPU on carbon emissions.

From an econometric point of view, the novelty of our paper is as follows: Firstly, we employ second-generation econometric techniques such as Pesaran’s (2015) cross-sectional dependence test, Pesaran (2007) unit root tests, Pedroni’s (2004) cointegration test and two-stage least square with cross-sectional seemingly unrelated regression (SUR) and panel corrected standard errors, panel generalised method of moments with cross-sectional SUR and panel corrected standard errors and generalised least square with correlation disturbances estimators. Since cross-sectional correlation of errors is the rule rather than the exception in panel data applications in economics, neglecting cross-sectional error dependency may have significant consequences. Connections within social and economic networks can result in cross-correlations in errors. This can occur due to a lack of common effects, geographic effects or the omission of common effects (Chudik and Pesaran 2013). Traditional panel estimators, such as fixed or random effects, can result in misleading inference and even inconsistent estimates, depending on the degree of cross-sectional dependence, and the degree to which the source of cross-sectional dependence (such as an unobserved common shock) is correlated with the regressors (Phillips and Sul 2003, 2007). Panel unit root tests may be influenced by the occurrence of correlations between the panels’ units. As a result, considerable size distortions may develop when unit root tests are used on cross-sectionally dependent panels (Andrews 2005). When the cross-sectional error dependency is minor or limited to a small number of cross-sectional units, the effect on classical estimators is negligible. When the source of cross-sectional dependency is connected to the regressors, the consistency of classical estimators is lost (O’connell 1998). Correlations between cross-sections and the modelling of cross-sectional error dependencies are crucial.

Typically, panel data models with short cross-sections and large time series employ a system of SUR equations to estimate cross-sectional dependence, which is then evaluated using generalised least squares techniques (Zellner 1962). The consistency of the SUR equation estimator is predicated on the premise that the source of cross-sectional dependency is unrelated to the regressors. If the time-series dimension is too small, $N > T$ renders the SUR equation approach infeasible. However, estimating a model with small $T$ and large $N$ which is not sufficiently large with generalised method of moments (GMM) as well as generalised least square (GLS) is considerably proven to be asymptomatically normal and consistent when the panel has a heterogeneous structure (Conley 1999) and both homogeneous and heterogeneous (Mark et al. 2005). We also employ the 2SLS in a dynamic simultaneous model with stationary and non-cointegrated variables because of its limiting features of an equation. It is demonstrated that when using a structural equation technique, the traditional issues of identification and estimation, rather than nonstationarity and cointegration, should be considered. Standard formulae for computing the asymptotic covariance of the 2SLS estimator and Wald-type test statistics are still sufficient approximations even though variables can be integrated (Hsiao 1997). Our sample presents a unique feature that is statistically important in estimating the medium-term impact of the variables of interest. The above arguments underpin our justification for employing the three estimation techniques for a robust conclusion of our findings.

Secondly, we utilise the EKC model and modify it to incorporate variables like foreign direct investment (FDI), urbanisation and renewable energy consumption as the theoretical proponents of our study.^1^ The inclusion of these variables would affirm the relevance of the STIRPAT model in the macro-environment context, as population growth and urbanisation, technological advancement and income accumulation are widely recognised as the underlying influence on the environment, either positively or negatively. More specifically, consumption and production-based emissions. Understandably, numerous studies have confirmed the relative importance of the STIRPAT model in evaluating the structural relationship between macroeconomic and environmental variables (Koop 1998; Koop and Tole 1999; Yu et al. 2021; Wang et al. 2022). Furthermore, we utilised data on eighteen (18) industrial economies with reliable EPU data. As of 2020, these 18 industrialised economies have a combined nominal GDP of 68.14 trillion, accounting for 80.44% of world GDP.^2^ However, their production and consumption patterns concerning energy demand, urban infrastructure, employment and labour participation rates, research and development, FDI inflows and technological innovation provide compelling evidence and an ideal sample for a major study of EPU vis-à-vis carbon emissions. More importantly, we present contributing evidence to shape modelling direction and contribute to the subject matter for academic and policy work.

The remainder of this paper is organised as follows: The next section encompasses the theoretical and empirical literature review. This is followed by the “Methodology and data” section, describing our methods, including data sources and estimation techniques. Finally, our results are presented and discussed in the “Results and discussion” section, followed by the conclusion and policy implications in the “Conclusion and policy implication” section.

## Literature review

### Theoretical underpinnings

Economists have utilised the environmental Kuznets curve (EKC) to study the phenomena of the environment and pollution since its introduction by Grossman and Krueger in 1991 (Panayotou 1993; Stern et al. 1996). The EKC is a hypothesis that elaborates on the nexus between per capita income and environmental degradation indicators. It is assumed that in the early stages of a country’s economic growth, environmental quality declines and pollution emissions burgeon. Nonetheless, the trend reverses at a certain level of per capita income. Therefore, at high-income levels, environmental quality begins to improve as economic production structures become more energy and environmentally efficient, so less pollution due to improved technologies (Guo 2015; Stern 2017). The implication is that per capita income is an inverted U-shaped function of emissions per capita or environmental effects.

Mathematically, the EKC can be expressed in the simplest form below:

1 $$y=a+bx+c{x}^{2}+\varepsilon$$

In Eq. (1), *y* represents the extent of environmental pressure, $x$ represents the level of output per capita in current form, and $\varepsilon$ represents the unobservable residual. Moreover, $a$ represents the constant term, and $b$ and $c$ are the parameter coefficients to be estimated, which reflect the income level impact on the quality of the environment.

The EKC suggests that countries’ environmental policies vary between low-income and high-income countries vis-à-vis the need to attract foreign direct investments in the context of international trade. Theoretically, in terms of FDI, two assumptions exist, namely the pollution halo effect (PHE) and the pollution haven hypothesis (PHH). One school of thought is that the process of globalisation (FDI and international trade) inherently means that polluting activities will inevitability find themselves concentrated in countries with weak environmental policies, often low-income ones. That is, stringent environmental measures in the northern hemisphere could lead to high prices for the industries situated in that region in relation to the prices charged by industries in the southern hemisphere – pollution policy as a source of comparative advantage. In other words, environmental regulation stringency can be characterised by a pollution haven hypothesis (OECD 2022; Shao et al. 2019; Solarin et al. 2017; Cole 2004; Singhania and Saini 2021; Smulders 2004).

On the other hand, the pollution halo effect (PHE) suggests that FDI inflows are usually environmentally friendly given that firms bring advanced technologies, cleaner and green energy usage, managerial expertise and ecological regulation compliance, among others (Liu and Xu 2021; Abid and Sekrafi 2021; Duan and Jiang 2021; Wang et al. 2019). In other words, multinational firms’ use of pollution abatement and renewable energy technologies leads to lower carbon emissions. Hence, FDI has a positive environmental impact. In contrast, as argued earlier, the assumption of the pollution haven hypothesis suggests that corporations move into countries with weak environmental regulations with outmoded practices to pollute the host country (Chen and Mkumbo 2020; Vitenu-Sackey 2020).

### Economic determinants of CO_2_ emissions

Numerous studies have offered different viewpoints on the determinants of carbon emissions at the country, sectoral and firm levels. Recent studies have focused on energy consumption, environmental pollution, economic growth, eco-innovation and economic policy uncertainty from separate perspectives or a combination of some of these factors (Adams et al. 2020; Chen and Mkumbo 2020; Fethi and Rahuma 2019; Jiang et al. 2019; Khan et al. 2020; Mensah et al. 2020; Wang et al. 2020a, 2020b). For example, Fethi and Rahuma (2019) assessed the impact of eco-innovation on carbon emission. They focused on the top 20 refined oil-exporting economies using dynamic, seemingly unrelated cointegration tests. Their findings suggest that research and development (R&D) investment, as a proxy of eco-innovation, negatively impacts carbon emissions in the long run. To buttress this, Khan et al. (2020) posit that renewable energy consumption, income, environmental innovation and trade have a stable relationship with carbon emissions – in their study conducted from 1990 to 2017 for G7 countries. Moreover, they confirmed that to abate carbon emissions in the long run, environmental innovation, exports and renewable energy consumption are significant factors. On the other hand, Chen and Mkumbo (2020) and Wang et al. (2020a) suggest otherwise. For example, Wang et al. (2020b, c, a) studied the relationship between carbon emissions and eco-innovation in China between 2004 and 2016 in a panel of 30 provinces. They contend that carbon emissions and eco-innovations are positively related such that environmental regulations and government policies mediate their relationship. Chen and Mkumbo (2020) studied the OECD as a sample, and their findings support Wang et al. (2020a).

### Economic policy uncertainty and CO_2_ emissions

Al-Thaqeeb and Algharabali (2019:2) define policy uncertainty as “the economic risk associated with undefined future government policies and regulatory frameworks.” In broad terms, such undefined future government policies (monetary and fiscal) and regulatory frameworks ultimately impact individual and firm-level decision-making. For example, companies can and do delay their spending and investment decisions by adopting a “wait and see attitude” due to the uncertainty that such policies create within the market. Within the literature, several works have been published which examine the impacts (often negative) of the economic policy uncertainty (proxied by some index measure) on households, businesses and economies (Bloom 2009, 2014; Baker et al. 2016). High uncertainty acts as a drag on households and businesses as they are pushed to behave in a more risk-averse or conservative manner for “fear of the unknown”. Global uncertainty in recent times has heightened political instability and economic policy volatility. This can quickly lead to lower aggregate demand (consumption) and thus lower economic growth and higher unemployment (Al-Thaqeb et al. 2020; Caggiano et al. 2017). For example, the events that have subsequently occasioned the ongoing COVID-19 pandemic from early 2020 to date are an excellent illustration of how policy uncertainty affects the overall society. Globally, several government decisions to impose lockdown measures and other non-pharmaceutical interventions to contain the spread of the virus, albeit successful, created significant uncertainty, which ultimately led to a slowdown in the global economy (Deb et al. 2021; Frempong et al. 2021; Dzator et al. 2021; Lau et al. 2020; Haider et al. 2020).

#### How does EPU influence carbon emissions?

Suppose economic policy uncertainty (EPU) is a significant driver of economic and investment activities. In that case, it could be argued that EPU will also impact energy consumption and ultimately carbon emissions since energy use is fundamental for economic growth. Within carbon markets and in the context of the global climate change debate, EPU affects carbon emissions via three channels: (1) firm [or country] innovation (increase or lessen efforts to reduce emissions), (2) share of fossil fuels in the energy mix, and (3) energy intensity (Yu et al. 2021). In times of uncertainty, the level of innovation in an economy serves as a stimulus for resilience. Firms’ green innovation can be driven by environmental uncertainties (Li et al. 2019) and economic policy uncertainties (Xu 2020). There is an assumption that pro-environmental innovation leads to technological advancement that propels process and product efficiencies and ultimately reduces carbon emissions (Khan et al. 2020; Gamso 2018). Numerous studies have documented that innovation and patents could be significantly affected by economic policy uncertainty (Bhattacharya et al. 2017; Chen et al. 2018), and this transcends to carbon emissions (Anser et al. 2021a, 2021b; Ling et al. 2015; Shahbaz et al. 2016).

In our opinion, we foresee economic policy uncertainty affecting carbon emissions through economic activities such as trade, stock market and investment, among others. Following the 2015 Paris Climate Agreement Accord, various countries have instituted measures to curb carbon emissions through investment in eco-friendly technologies (Wang et al. 2020a). Some empirical works have posited that eco-friendly technological innovations significantly diminish carbon emissions, strengthen economies and improve firm performance (Khan et al. 2020; Zhang et al. 2017). Economic efficiency stems from the capacity of firms to produce goods and services through technology adoption and implementation. This combination of resource efficiency and reducing costs associated with the environment is termed environmental innovation. Therefore, the expectation is that energy intensity due to R&D and patents could significantly impact carbon emissions. As theoretically and empirically tested, renewable energy consumption and carbon emissions are inversely related (Mensah et al. 2020; Vitenu-Sackey 2020; Wang et al. 2020b) as it has similar characteristics of ecological innovation. Conversely, cleaner and pure energy sources from renewable energy technologies ensure the sustainable energy supply of future and current energy needs.

**Fig. 1:**
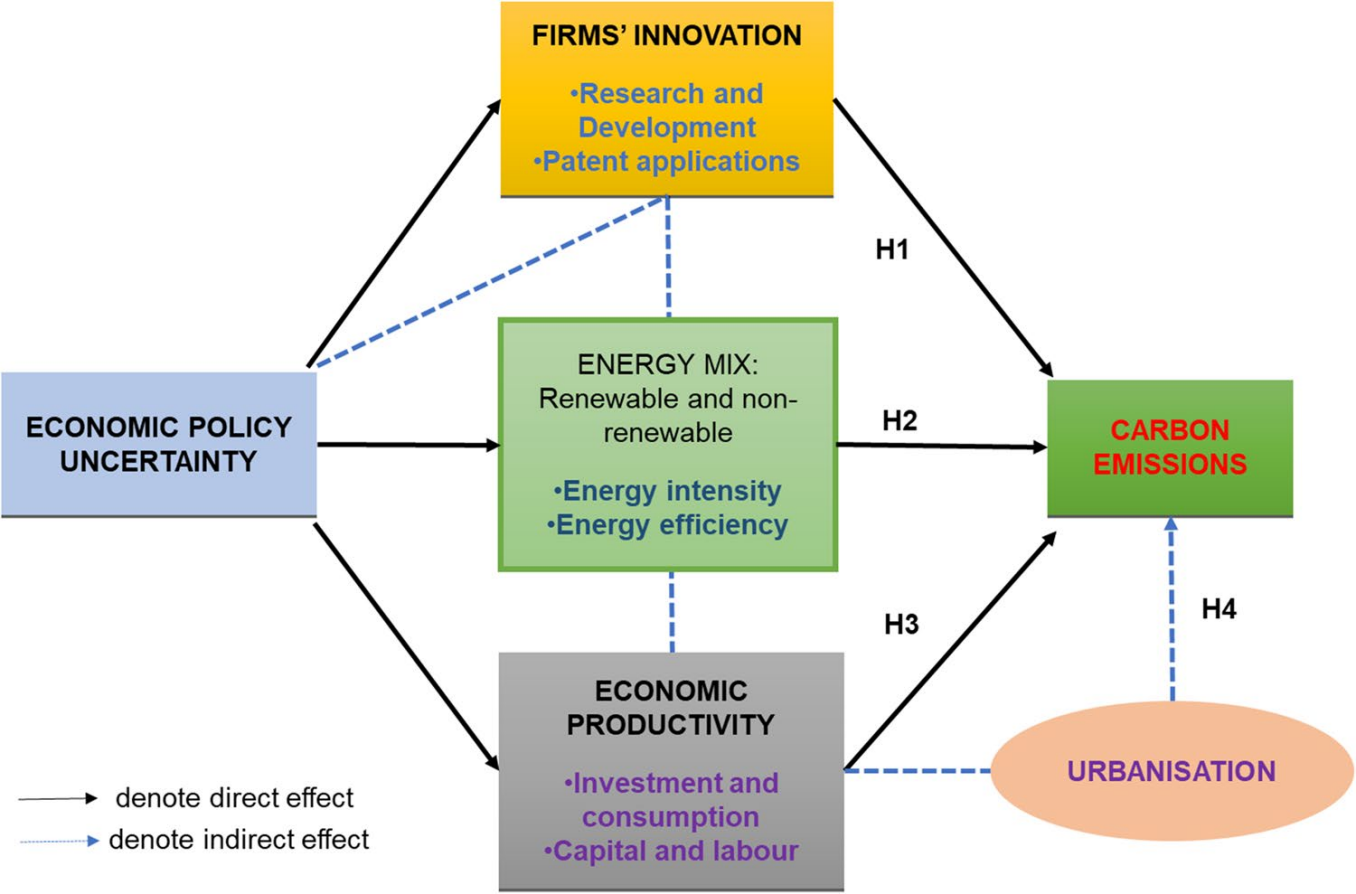
Conceptual framework of the relationship between economic policy uncertainty and CO_2_ emissions

Table 1 below provides a broad summary of some recent literature on carbon emissions, innovation and EPU. However, we note here that the conclusions in the existing literature on the relationship between EPU and carbon emission are not entirely conclusive. There is thus the need for further investigation using other functional econometric forms and new data variables as additional controls. This is where our paper adds to the debate by filling some of the voids. Figure 1 is our conceptual framework, illustrating the relationship between economic policy uncertainty and CO_2_ emissions. Based on this and the underlying literature, we make the following hypotheses:

- *H1: High EPU causes firm innovation, including in carbon abatement technologies, to stall.*
- *H2: High EPU reduces the need for stringent environmental regulations/protections, which increases carbon emissions.*
- *H3: High EPU leads to delays in investment and consumption, including in carbon abatement technologies.*

**Table 1:** Summary of some recent literature on carbon emissions, innovation and EPU

Author(s)	Methodology, sample and context	Findings
Wang et al. (2022)	•Stochastic Impacts by Regression on Population, Affluence and Technology (STIRPAT) model based on GMM estimations •Period: 1970–2018 •137 countries	•EPU would bring about more carbon emissions •Effect of EPU on air pollution in OECD countries is lower than in non-OECD ones (higher levels of economic development reduce the adverse environmental effect of EPU) •Higher globalisation and more international trade weaken the effect of EPU on CO_2_ emissions
Nakhli et al. (2022)	•Bootstrap Rolling approach •Country: USA •Period: 1985–2020	•Bidirectional causality between CO_2_ emissions and EPU in the USA
Yu et al. (2021)	•STIRPAT model (Stochastic Impacts by Regression on Population, Affluence and Technology) via a two-way fixed effect model •Country: China •Period: 2008–2011	•Significance of fuel mix and energy intensity channels; however, innovation channel is not •Chinese firms prefer to use cheap and dirty fossil fuels to react to the rising EPU
Syed and Bouri (2021)	•Bootstrap ARDL •Country: USA •Period: 1985–2019	•High EPU intensifies CO_2_ emissions (environmental degradation) in the short run •High EPU betters environmental quality in the long run
Appiah-Otoo (2021)	•IV-GMM model plus OLS estimator •20 countries •Period: 2000–2018	•EPU has an insignificant negative effect on RE growth •No evidence of causality between EPU and RE growth
Adams et al. (2020)	•Panel Pooled Mean Group-Autoregressive Distributed lag model (PMG-ARDL) •Countries: 10 resource-rich countries with high geopolitical risk •Period: 1996–2017	•Significant long-run association between EPU and CO_2_ emissions
Pirgaip and Dinçergök (2020)	•Bootstrap panel Granger causality test •G7 countries •Period: 1998–2018	•Uni-directional causality from EPU to CO_2_ emissions in the USA and Germany •Uni-directional causality from EPU to energy consumption in Japan •Uni-directional causality from EPU to energy consumption and CO_2_ emissions in Canada, the USA and Italy
Adedoyin and Zakari (2020)	•Autoregressive distributed lag model (ARDL) bound test •Country: United Kingdom •Period: 1985–2017	•Uni-directional causality from CO_2_ emissions to EPU in the United Kingdom •Uni-directional causality from energy use to EPU
Wang et al. (2020b)	•Autoregressive distributed lag (ARDL) model •Country: USA •Period: 1960–2016	•World Uncertainty indices are positively associated with CO_2_ emissions in the long run (higher EPU uncertainty in the previous year in the USA leads to higher CO_2_ emissions in the current period) •Per capita income increases CO_2_ emissions in the long run
Khan et al. (2020)	•Advanced panel data estimation techniques/panel cointegration methodologies •Period: 1990–2017 •Coverage: G7 countries	•Imports and income have a long-run positive impact (increase with) on consumption-based CO_2_ emissions •Exports, environmental innovation and RE consumption are negatively related to consumption-based CO_2_ emissions
Jiang et al. (2019)	•Quintile parametric test of Granger causality •Country: USA •Period: 1985–2017	•Granger causality from EPU to CO_2_ emissions •Causality applies in the industrial sector, residential sector, electric power sector and transportation sector, except for the commercial sector
Fethi and Rahuma (2019)	•Panel time-series framework •Coverage: top 20 refined oil-exporting countries •Period: 2007–2016	•Eco-innovation (R&D) has a negative and significant long-run effect on CO_2_ emissions
Anser et al. (2021a, 2021b)	•Panel data analysis – using FMOLS and DOLS •Coverage: 5 emerging economies •Period: 1995–2015	•Economic policy uncertainty and non-clean or renewable energy consumption surge carbon emissions but renewable energy thwarts carbon emissions
Ahmad et al. (2021b, a)	•Panel study using DCCE •Coverage: 28 Chinese provinces •Period: 1998–2016	•EKC, PHE and PHH are not entirely connected to the extent of development •Income levels and foreign direct investment inflow are related to ecological quality heterogeneously
Abbasi and Adedoyin (2021)	•Time-series study using Novel dynamic ARDL simulation method •Coverage: China •Period: 1970–2018	•Energy consumption directly affects environmental quality •Economic policy uncertainty does not substantially contribute to carbon emissions •Economic growth and energy intensity are the short- and long-run drivers of carbon emissions •There is an aggregation bias when dealing with economic indicators and environmental quality
Anser et al. (2021a, 2021b)	•Panel data analysis using the PMG-ARDL method •Coverage: top ten carbon emitters •Period: 1990–2015	•Policy uncertainty negatively relates to carbon emission in the short run but is progressive in the long run

## Methodology and data

### Estimation approach

On the theoretical proponent of the EKC model, we propose the model below to rely on for our empirical study. This model follows the assumptions of Fethi and Rahuma (2019), De Vita et al. (2015), Kapusuzoğlu (2014) and Pao and Tsai (2011).

2 $${lnCO2}_{i,t}= {\beta }_{0}+{\beta }_{1} {lnY}_{i,t}+{\beta }_{2} {ln{Y}^{2}}_{i,t}+{\beta }_{3} {EU}_{i,t}+{\beta }_{4} {R\&D}_{i,t}+ {\varepsilon }_{i,t}$$

where lnCO2 denotes the natural logarithm of carbon emissions, $lnY$ and ${lnY}^{2}$ are the natural logarithm of income per capita and squared income per capita in real terms, $lnEU$ denotes energy consumption, $lnR\&D$ denotes research and development used to proxy ecological innovation, and $\varepsilon$ represents the error term.

We modify this model to include a policy shock (economic policy uncertainty), which reflects Jiang et al. (2019) and Adams et al. (2020) assertions that economic policy uncertainty has a connection with carbon emissions. On the other hand, we also follow Mensah et al. (2020), Vitenu-Sackey (2020) and Chen and Mkumbo (2020) to include urbanisation, renewable energy consumption and FDI into the model. The inclusion of FDI in the model is premised on the proponent of the pollution halo effect (PHE) and pollution haven hypothesis (PHH). Therefore, we propose this model:

3 $$CO2=f \left(EPU+ EINT+PT+R\&D+GDPCAP+GDPCAP2+FDI+RE+URP\right)$$

In Eq. (2), CO_2_ represents carbon emission, EPU represents economic policy uncertainty, patent registration denotes PT, research and development expenditure denotes R&D, and energy intensity denotes EINT. GDPCAP represents economic growth, GDPCAP2 represents the diminishing returns of economic growth, FDI represents a foreign direct investment, RE represents renewable energy consumption, and URP represents urbanisation.

To perform an econometric analysis on the theoretical model proposed, we construct the econometric model below:

4 $${CO2}_{i,t}= {\beta }_{0}+{\beta }_{1} {EPU}_{i,t}+{\beta }_{2} {EINT}_{i,t}+{\beta }_{3} {PT}_{i,t}+{\beta }_{4} {R\&D}_{i,t}+{\beta }_{5} {GDPCAP}_{i,t}+ {\beta }_{6}{ GDPCAP2}_{i,t}+{\beta }_{7}{ RE}_{i,t}+{\beta }_{8}{ FDI}_{i,t}+{\beta }_{9}{ URP}_{i,t}+ {\varepsilon }_{i,t}$$

In Eq. (3), CO_2_ represents carbon emission, EPU represents economic policy uncertainty, PT represents patent registration, R&D represents research and development expenditure, and EINT represents energy intensity. GDPCAP represents economic output, GDPCAP2 represents the higher economic output, FDI represents foreign direct investment, RE represents renewable energy consumption, and URP represents urbanisation. ${\beta }_{0}$ represents the constant term or intercept of the slope, ${\beta }_{1}$ to ${\beta }_{7}$ represent the parameters’ coefficients to be estimated, $\varepsilon$ represents the error term, $i$ represents the cross-section of 18 countries, and $t$ represents the period from 2005 to 2018.

Conventionally, econometric analysis requires preliminary tests to ensure data validity and reliability. Therefore, prior to the estimation of the parameters, we performed some tests, including (1) unit root test, (2) cross-sectional dependence and homogeneity test, (3) cointegration test, (4) correlation matrix and (5) sample adequacy test. In our quest to ascertain the stationarity status of the data series, we employed the panel unit root tests of Pesaran (2007), namely the cross-sectionally augmented panel unit root test (CIPS) and cross-section augmented Dickey-Fuller (CADF). Specifically, these tests are reliant due to their capability to provide more accurate and consistent results.

Subsequently, we utilised the cross-sectional dependence test of Pesaran (2015) to ascertain the cross-sectional dependence of the residuals and heterogeneous slopes among the panels. Pesaran’s (2015) cross-sectional dependence test unravels variables with weak cross-sectional dependence, which implies that it is statistically robust in that context. After the cross-sectional dependence test, we checked for the long-run relationship among the variables using the Kao (1999) and Pedroni (2004) tests. The independent variables having high correlation coefficients with the dependent variables could signal multicollinearity issues in the model. Therefore, to ensure no multicollinearity, we computed a correlation matrix to check for multicollinearity and ascertain the correlation coefficients and signs of the independent variables against the dependent variable. We performed a sample adequacy test to rely on the sample selected for the study firmly. In that context, we performed the KMO and Bartletts’ test of sphericity.

After careful satisfactory preliminary tests, we subsequently performed the long-run parameter estimations. The estimations are done with three estimators as follows:

1. two-stage least square (2SLS) with cross-sectional SUR and panel corrected standard errors (PCSE),
2. panel generalised method of moments (GMM) with cross-sectional SUR and panel corrected standard errors, and lastly,
3. generalised least square (GLS) with correlation disturbances for a robust conclusion.

The two-stage least square with cross-sectional SUR (PCSE) and generalised method of the moment with cross-sectional SUR (PCSE) is described as the best estimators due to their functions of incorporating endogenous regressors and efficiency. These resolve the issues of cross-sectional endogeneity, simultaneity and heterogeneity in the panels (Neal 2015). Regarding the generalised least squares (GLS) with correlation disturbances estimator, Koreisha and Fang (2001) contend that the estimator corrects inefficient parameters and resolves serial correlation and autocorrelation in the panels. To robustly conclude our findings from the two-stage least square and GMM estimators, we performed a cross-sectional dependence test to confirm the assumption that there is evidence of cross-sectional dependence in the panels. Moreover, an instrument validity test was performed to reject the assumption of weak instruments as well as autocorrelation.

Finally, we perform a homogenous causality test of Dumitrescu and Hurlin (2012). In this regard, we tend to ascertain the direction of causality between the dependent and the independent variables. Moreover, the causal relationships established would offer policy direction (Shahbaz et al. 2012).

### Data

We utilised data for 18 countries based on information gathered on EPU from www.policyuncertainty.com. Specifically, the data on EPU is available for only 24 countries. However, we relied on 18 advanced economies due to consistent data availability. These were Australia, China, Canada, Russia, Korea, Mexico, Greece, the United Kingdom, the Netherlands, the USA, France, Chile, Germany, Ireland, Spain, Italy, Sweden and Japan.

Also, while the data on EPU starts from 2000, most countries did not have available data between 2000 and 2004. Therefore, we chose the study period from 2005 to 2018. Our dependent variable is CO_2_ emissions, and the independent variables are economic policy uncertainty, energy intensity, research and development, and patent registrations as a proxy to measure technological innovation. However, we used some variables as control variables: foreign direct investment, renewable energy consumption, gross domestic product per capita, higher gross domestic product per capita and urbanisation (urban population) (see Appendix Table 10 and 11 for more details).

**Fig. 2:**
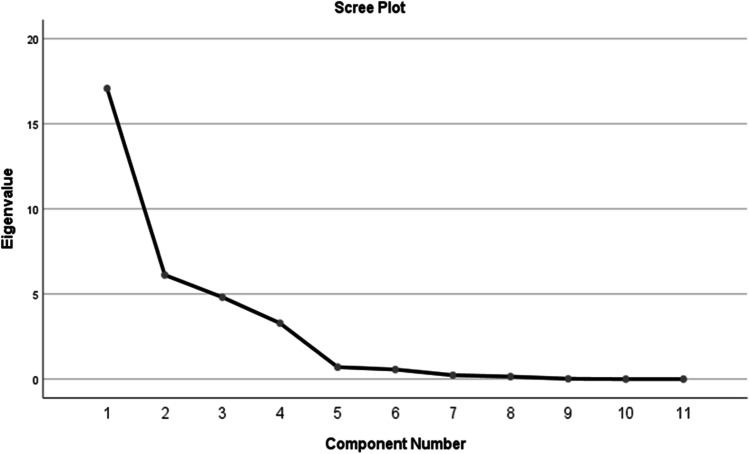
Scree plot of eigenvalue extracted

To ensure the adequacy of our sample, we performed KMO and Bartlett’s test, and the results are presented in Table 2. Evidence suggests that the sample used in our study is adequate as the variance explained amounted to 72% of an eigenvalue greater than 1. Figure 2 also confirms the results of the sample adequacy test depicting the scree plot.

**Table 2:** Sample adequacy test

KMO and Bartlett’s test		
Kaiser–Meyer–Olkin measure of sampling adequacy		0.720
Bartlett’s test of sphericity	Approx. chi-square	3308.081
	df	45
	Sig	0.000

## Results and discussion

### Findings

Table 3 presents the descriptive statistics. Highlighting the vital statistics, we observed that CO_2_ had a mean of 2.069 and a standard deviation of 0.464. Averagely, energy intensity has been reducing in the sampled countries, considering the mean value of − 12.574 and a standard deviation of 0.683. To account for the other variables, we observed that GDP_CAP had the highest mean value of 10.396 and a standard deviation of 0.460. The second variable with highest mean value is FDI (9.754) and a standard deviation of 2.562 while PT (mean = 6.311, standard deviation = 2.153), R&D (mean = 1.678, standard deviation = 3.523) and EPU (mean = 4.808, standard deviation = 0.550) – see Table 3 for more details. We could elucidate GDPCAP as the variable with the highest mean value based on aggregate demand attributing to minimal government expenditure, increased investment to surge production and reduction in interest rates, among others, resulting in increased incomes.

**Table 3:** Descriptive statistics of the variables

	Mean	Median	Max	Min	Std. dev	Skewness	Kurtosis	Jarque–Bera	Obs
CO_2_	2.069	2.140	2.965	1.194	0.464	0.069	2.121	8.307**	252
EINT	− 12.574	− 12.695	− 9.936	− 14.564	0.683	0.967	5.108	85.876***	252
PT	6.311	6.597	9.868	1.674	2.153	− 0.336	2.283	10.156**	252
R&D	1.678	0.661	13.045	− 1.173	3.523	2.358	7.010	402.293***	252
EPU	4.808	4.800	6.354	2.728	0.550	− 0.096	3.574	3.841	252
GDPCAP	10.396	10.515	11.345	8.525	0.460	− 1.351	5.095	122.713***	252
GDPCAP2	3.223	3.243	3.368	2.920	0.073	− 1.430	5.390	145.911***	252
FDI	9.754	10.287	13.090	0.000	2.562	− 2.598	10.438	864.399***	252
RE	2.570	2.074	12.596	− 0.673	2.518	3.160	12.802	1428.198***	252
URP	− 0.131	0.031	1.356	− 6.098	0.883	− 2.318	13.089	1294.569***	252
EI	0.000	− 0.177	3.861	-2.913	1.000	0.967	5.108	85.881***	252

Table 4 presents the cross-sectional dependence and unit root test results. We observed from the outcome that all the variables were stationary with both CIPS and CADF tests at the first difference I(1). Therefore, we reject the assumption that the variables are non-stationary and hence have a unit root at the 1% and 5% significance levels, respectively. Nonetheless, all the variables except URP exhibited cross-sectional dependence, implying that their residuals or error terms could not depict dependency in the individual panels. This revelation requires the use of estimators that could reliably resolve that issue.

**Table 4:** Cross-sectional dependence and unit root tests

	CD	CIPS I(0)	CIPS I(1)	CADF I(0)	CADF I(1)
CO_2_	44.481***	− 1.623	− 3.186***	0.338	− 5.744***
EINT	44.594***	− 2.019	− 3.371***	− 1.202	− 6.466***
PT	44.471***	− 2.246**	− 3.999***	− 1.218	− 2.447**
R&D	17.920***	− 2.692***	− 3.276***	− 3.821***	− 6.094***
EPU	44.410***	− 2.835***	− 4.571***	− 4.381***	− 11.135***
GDPCAP	44.596***	− 1.833	− 2.878***	− 0.653	− 2.292**
GDPCAP2	44.598***	− 1.869	− 2.880***	− 0.620	− 4.555***
FDI	42.412***	− 3.014***	− 4.484***	− 2.118**	− 7.943***
RE	38.836***	− 1.088	− 3.566***	− 2.858**	− 4.381***
URP	0.025	− 1.346	− 3.067***	1.418	− 5.283***

^***^1% significance level

^**^5% significance level

*CD*, cross-sectional dependence

CIPS & CADF = Pesaran unit root tests. I(0) = level form. I(1) = first difference. (See appendix for variables description)

The outcome of a cointegration analysis to check the long-run relationship amid the exogenous and the endogenous variables using Kao (1999) and Pedroni (2004) tests is presented in Table 5. From the results, we can firmly confirm that the variables are cointegrated at 1% and 5% significance levels.

**Table 5:** Cointegration test

				Weighted		
	Statistic	Prob	Sig	Statistic	Prob	Sig
Panel v-statistic	− 4.841	1.000		− 6.687	1.000	
Panel rho-statistic	9.849	1.000		6.852	1.000	
Panel PP-statistic	− 12.613	0.000	***	− 16.452	0.000	***
Panel ADF-statistic	− 7.546	0.000	***	− 7.523	0.000	***
Alternative hypothesis: individual AR coefficients (between-dimension)						
	Statistic	Prob				
Group rho-statistic	10.536	1.000				
Group PP-statistic	− 25.256	0.000	***			
Group ADF-statistic	− 9.425	0.000	***			
Kao residual cointegration test						
	*t*-statistic	Prob	Sig			
ADF	− 5.276	0.000	***			

^***^1% significance level

^**^5% significance level

After checking for the long-run relationship amid the exogenous and endogenous variables, we further checked for multicollinearity in the proposed model with the aid of a correlation matrix. The correlation matrix unravels two statistical information: (1) correlation coefficients and (2) multicollinearity. In that regard, we present the outcome of the correlation matrix in Table 6. We observed that EINT, RE and URP are negatively correlated with the CO_2_ variable. In contrast, PT, R&D, FDI, EPU, GDPCAP and GDPCAP2 showed a positive correlation with the CO_2_ variable – but R&D, FDI and URP showed insignificant correlations. On the other hand, we found no multicollinearity in our proposed model as the variable with the highest correlation coefficient is GDP_CAP, followed by GDP_CAP2 with 0.454 and 0.453 coefficients, respectively. According to Sun et al. (2002) and Mensah et al. (2020), independent variables with correlation coefficients of − / + 0.70 and above are considered highly correlated with the dependent variable – hence, multicollinearity is present in that model.

**Table 6:** Correlation matrix

Probability	CO_2_	EPU	EINT	PT	R&D	GDP_CAP	GDP_CAP2	FDI	RE	URP
CO_2_	1									
EPU	0.120*	1								
EINT	− 0.114*	− 0.129**	1							
PT	0.401***	0.197**	− 0.187**	1						
R&D	0.067	0.204***	0.690***	0.112*	1					
GDPCAP	0.454***	0.204***	− 0.880***	0.387***	− 0.539***	1				
GDPCAP2	0.453***	0.202***	− 0.880***	0.383***	− 0.544***	1.000***	1			
FDI	0.035	0.141**	0.124**	0.077	0.206***	− 0.070	− 0.074	1		
RE	− 0.281***	0.045	0.531***	0.048	0.614***	− 0.593***	− 0.601***	0.228***	1	
URP	− 0.096	− 0.126**	0.122**	− 0.058	0.005	− 0.189**	− 0.195**	0.088	0.376***	1

^***^1% significance level

^**^5% significance level

^*^10% significance level

### Long-run estimations

As highlighted in the methodology section, we employed three robust estimators for our long-run estimations to resolve problems of heterogeneity, serial correlation, autocorrelation, endogeneity and simultaneity in the panels. We observed good and reliable outcomes in an account of the estimators’ fitness, which implies that our models fit for inference. Specifically, we observed that the 2SLS and GMM (CSUR-PCSE) reported *r*-squared of 0.585 and 0.571 – symbolising 58.5% and 57.1% variance of the dependent variable explained by the independent variables (Table 7). Moreover, the models’ instruments are robust, considering the J-statistics and its probability – showing *p*-values greater than 0.05 (Table 7). On the other hand, we observed cross-sectional dependence in the models where all three cross-sectional dependency tests produced significant values, particularly at a 1% significance level. Suffice to say, we could not substantiate evidence of autocorrelation in the model. Notably, we employed very reliable and efficient methodologies due to their function in unravelling the heterogeneity and cross-sectional dependence among the slope parameters.

**Table 7:** Long-run parameter estimations

	**2SLS-CSUR PCSE**	**GMM-CSUR PCSE**	**GLS-COR**
EPU	− 0.051	− 0.051	− 0.041
	(− 1.724)*	(− 1.724)***	(− 9.987)***
EINT	0.724	0.724	0.735
	(24.874)***	(24.874)***	(76.49)***
PT	0.013	0.013	0.013
	(2.746)**	(2.746)**	(10.26)***
R&D	0.026	0.026	0.024
	(7.542)***	(7.542)***	(21.84)***
GDPCAP	1.742	1.742	3.413
	(22.159)***	(22.159)***	(9.72)***
GDPCAP2	− 2.080	− 2.080	− 12.789
	(− 7.305)***	(− 7.305)***	(− 5.76)***
FDI	− 0.001	− 0.001	− 0.002
	(− 0.176)	(− 0.176)	(− 2.44)**
RE	− 0.033	− 0.033	− 0.034
	(− 5.963)***	(5.963)***	− 22.19)***
URP	0.052	0.052	0.043
	(4.461)***	(4.461)***	(18.43)***
Constant			17.249
			(4.96)***
*R*-squared	0.585	0.585	
Adjusted *R*-squared	0.572	0.572	
J-statistic		0.439	
Prob (J-statistic)	0.507	0.507	
Wald chi2			72,285.00***
Autocorrelation			No
Obs	252	252	252

^***^1% significance level

^**^5% significance level

^*^10% significance level

The number in parentheses are the standard errors. See appendix for variables description

The outcome of all the three estimators showed similar results regarding coefficients and significance. In particular, the two-stage least square and generalised method of moment with cross-sectional SUR and panel corrected standard errors (PCSE) estimators produced the same results regarding the coefficients except for GLS, which only differs. More importantly, we realised that foreign direct investment exhibited a negative relationship with carbon emissions in all the three estimators, but only the GLS estimator produced significant coefficients.

**Fig. 3:**
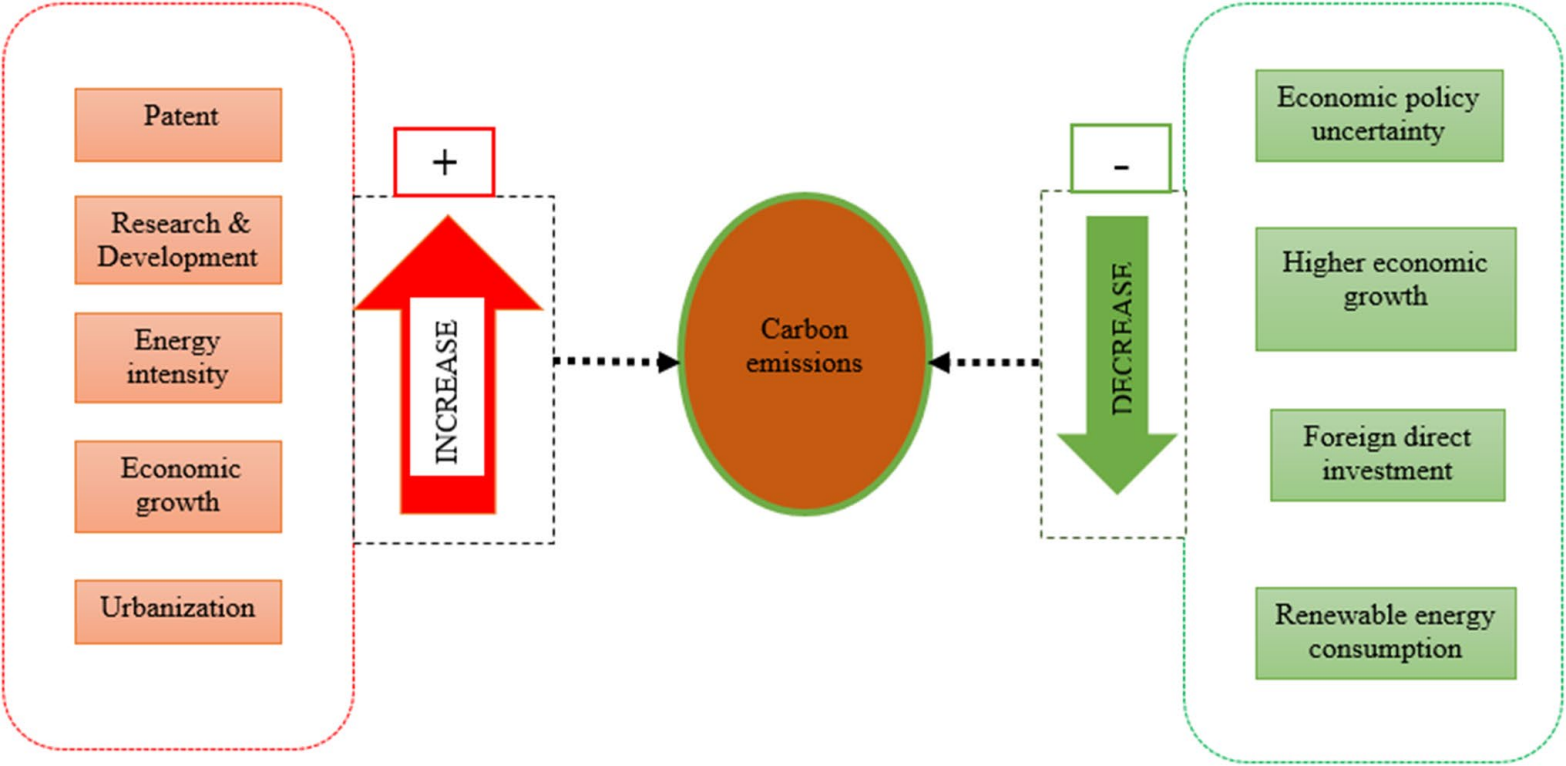
Pictorial display of findings showing the modelled relationships

Specifically, we observed a significant positive relationship between technological innovation, research and development, energy intensity and carbon emissions. However, economic policy uncertainty disproportionally leads to carbon emissions, affirming a negative relationship (Fig. 3). Evidence from all the three estimators suggests that a percentage point increase in economic policy uncertainty reduces carbon emission by 0.051%, 0.041% and 0.014% at the 10% and 1% significance levels (Table 7). Also, we observed that energy intensity (EINT) increases carbon emissions by 0.724% and 0.735%, with a percentage point increase at the 1% significance level for all the three estimators (Table 7). Also, patent (PT) and research and development (R&D) lead to an increase in carbon emissions by 0.013%, 0.024% and 0.026% at both the 1% and 5% significance levels (Table 7).

With regard to economic growth’s impact on carbon emissions, we observed that economic growth significantly contributes to carbon emissions. However, the carbon emission reduces at an optimal level of growth, implying that our sample’s EKC hypothesis indicates a U-inverted relationship between economic growth and carbon emissions. Moreover, it also indicates that countries in our sample are particular about improving their environmental quality. Therefore, they resort to cleaner and environmentally friendly production methods in the long run when their output level surges. In particular, a percentage point in economic growth significantly increases carbon emissions by 1.742%, 2.964%, 3.413% and 5.184% at a 1% significance level, respectively, for all three estimations (Table 7). Nevertheless, when economic growth reaches an optimal level, then a percentage point increase reduces carbon emissions by 2.080%, 8.871%, 12.789% and 23.154% at a 1% significance level.

Considering the impact of urbanisation on carbon emissions, we also consistently observed a positive relationship among them. Specifically, a percentage point increase in urban population growth rate leads to an increase in carbon emission, which aggravates efforts to reduce carbon emissions by 0.052%, 0.030%, 0.043% and 0.025% at the 1% and 5% significance levels (Table 7). Meanwhile, it is evident in our samples that foreign direct investment and carbon emission are inversely related. This implies that foreign direct investment has pollution halo effects in the sampled countries rather than pollution haven hypothesis effects. In particular, a percentage point increase in foreign direct investment reduces carbon emission by 0.002% at 1% and 5% significance levels (Table 7).

To capture the heterogeneous characteristics of our sample, we classified the countries into two groups: high polluters and low polluters (see Appendix Table 11A for the classification of countries). We used CarbonBrief’s classification of countries based on cumulative carbon emissions from fossil fuel consumption from 1850 to 2021.^3^ The outcome of our findings is reported in Table 8. Our findings indicate that the impact of economic policy uncertainty, energy intensity, R&D expenditure, foreign direct investment, urbanisation, renewable energy consumption and patent applications, except for gross domestic product per capita and its quadratic term, which suggests symmetric relationships for both low- and high-polluting countries. In other words, the symmetric connections between GDP and GDP2 demonstrate the presence of the EKC hypothesis in both low and high polluters. Surprisingly, we discovered that renewable energy consumption and R&D have a positive and considerable influence on carbon emissions in high-polluting countries with high economic policy uncertainty. This finding supports our hypothesis that “High EPU causes firm innovation, including in carbon abatement technologies, to stall” and raises the cost of technology deployment. Furthermore, regardless of country characteristics, patent applications as a proxy for technological innovation appear to have no influence on carbon emissions, showing that not all innovation contributes to carbon emissions and that ecological innovation should be used to measure innovation impact.

**Table 8:** Heterogeneous panel analysis

	**High polluters**		**Low polluters**	
DEP = CO_2_	2SLS-CSUR PCSE	GMM-CSUR PCSE	2SLS-CSUR PCSE	GMM-CSUR PCSE
EPU	− 0.202***	− 0.202***	− 0.004	− 0.004
	(− 12.705)	(− 12.705)	(− 0.519)	(− 0.519)
EINT	0.838***	0.838***	0.105**	0.105**
	(34.658)	(34.658)	(2.503)	(2.503)
PT	− 0.005	− 0.005	0.006	0.006
	(− 1.614)	(− 1.614)	(0.911)	(0.911)
R&D	0.019***	0.019***	− 0.075***	− 0.075***
	(4.464)	(4.464)	(− 4.202)	(− 4.202)
GDPCAP	2.314***	2.314***	0.928***	0.928***
	(31.091)	(31.092)	(10.656)	(10.656)
GDPCAP2	− 3.243***	− 3.243***	− 1.817***	− 1.817***
	(− 12.670)	(− 12.670)	(− 8.371)	(− 8.371)
FDI	0.001	0.001	− 0.016***	− 0.016***
	(0.446)	(0.446)	(− 7.064)	(− 7.064)
RE	0.011**	0.011**	− 0.205***	− 0.205***
	(2.746)	(2.746)	(− 18.687)	(− 18.687)
URP	0.038***	0.038***	− 0.017**	− 0.017**
	(6.828)	(6.828)	(− 1.938)	(− 1.938)
Constant				
*R*-squared	0.969	0.970	0.821	0.821
Adjusted *R*-squared	0.968	0.968	0.809	0.809
J-statistic		0.031		3.458
Prob (J-statistic)	0.860	0.860	0.063	0.063
Instruments	10	10	10	10
Obs	126	126	126	126

^***^1% significance level

^**^5% significance level

^*^10% significance level

The number in parentheses are the standard errors. See appendix for variables description

It is worth emphasising that our data show that urbanisation, which has a large economic impact on most economies, is favourably associated with carbon emissions in high-polluting nations but negatively and significantly in low-polluting countries. According to these findings, most urban centres in low-polluting countries are less densely inhabited than high-polluting ones. To put it another way, rising demand for economic products and services such as energy, transportation and road infrastructure, among many others, contribute tremendously to emissions, particularly in times of severe economic policy uncertainty.

After considering the foreign direct investment, we can conclude that, while it is considerable, its impact on carbon emissions is not significant in high-pollution countries but is significant and contributes negatively to carbon emissions in low-pollution countries. This conclusion shows that foreign direct investment in low-pollution countries is ecologically responsible, which may support the pollution halo effect hypothesis (PHE). On the other side, it could indicate that those countries’ economic structures are dominated by the service sector and do not appear to be highly invested in high-energy-demanding businesses.

Notably, it is clear that economic policy uncertainty has no effect on carbon emissions in low-emissions countries, while economic growth – proxied by per capita incomes – contributes significantly to carbon emissions. In contrast, once income hits a particular threshold or optimal level, subsequent increases in income per capita cut emissions due to the implementation of sound environmental regulations and energy-efficient technology, as our data suggests (the quadratic term of GDP, GDP2).

We performed both D-H homogeneous and Granger causality tests to understand the causal relationship between carbon emission and the independent variables. The outcome of the tests is presented in Table 9. From the outcome, we observed that research and development (R&D) as a proxy of ecological innovation homogenously causes carbon emissions in a uni-directional way. Also, economic growth (GDP_CAP) and higher economic growth (GDP_CAP2) in a uni-directional way and homogeneously cause carbon emissions. Meanwhile, carbon emissions and renewable energy consumption (RE) have a uni-directional causal relationship.

**Table 9:** Panel causality tests

Pairwise Dumitrescu Hurlin panel causality tests			
Null hypothesis	W-stat	Sig	Causality
EINT → CO_2_	3.870		No
CO_2_ → EINT	4.066		No
PT → CO_2_	3.278		No
CO_2_ → PNT	3.262		No
R&D → CO_2_	5.516	**	Yes
CO_2_ → R&D	3.170		No
EPU → CO_2_	2.222		No
CO_2_ → EPU	2.943		No
GDP_CAP → CO_2_	5.097	**	Yes
CO_2_ → GDPCAP	4.348		No
GDP_CAP2 → CO_2_	5.109	**	Yes
CO_2_ → GDPCAP2	4.356		No
FDI → CO_2_	5.495	**	Yes
CO_2_ → FDI	2.062		No
RE → CO_2_	2.951		No
CO_2_ → RE	53.680	***	Yes
URP → CO_2_	4.789	**	Yes
CO_2_ → URP	4.141		No

^***^1% significance level

^**^5% significance level

^*^10% significance level

See appendix for variables description

Invariably, the causal relationship between economic growth, diminishing returns of economic growth and carbon emissions produced by the test showed the same direction of causality. Interestingly, in the Granger causality test, renewable energy consumption causes carbon emissions in a uni-directional that differs from the D-H causality test results. In particular, we observed a uni-directional causal relationship from carbon emissions to renewable energy consumption while foreign direct investment homogeneously causes carbon emissions in a uni-directional way. More importantly, we realised that economic growth and higher economic growth consistently showed a one-way causal relationship with carbon emissions. This implies that economic growth’s intensity on carbon emission is significant in our sample and is consistent with Fethi and Rahuma (2019) and Mensah et al. (2020).

### Discussion

Our study presents interesting findings: We observed a positive and significant impact of ecological innovation on carbon emission and a negative impact of economic policy uncertainty on carbon emissions. These findings oppose the studies of Fethi and Rahuma (2019) and Khan et al. (2020). Both studies contend that there is an inverse relationship between technological innovation and carbon emissions. Innovations created with the environment in mind tend to reduce pollution, thereby abating carbon emissions. However, some empirical studies suggest that economic policy uncertainty adversely impacts innovation, specifically patent applications. Therefore, economic policy uncertainty influences innovation proportionally related to carbon emissions (Bhattacharya et al. 2020; U. Bhattacharya et al. 2017; Chen and Mkumbo 2020; Chen et al. 2018).

Moreover, a high rise in carbon emissions requires a decrease in the level of economic policy uncertainty because an increase in carbon emission depicts an increase in production and consumption without restraints of economic policies. In particular, our findings support the study of Chen and Mkumbo (2020), where they established that energy intensity, patent, research and development and carbon emissions are positively and significantly related. In view of this, the increasing carbon emission rate requires accelerated ecological innovation to curtail the menace (Wang et al. 2020a).

Energy consumption (intensity) has been identified by many studies as a positive driver of carbon emissions, whereas increases in energy demand without recourse to renewable energy – surges carbon emission. We observed this positive impact in our findings in support of studies by Adams et al. (2020), Chen and Mkumbo (2020), Mensah et al. (2020) and Fethi and Rahuma (2019). Ultimately, renewable energy reduces carbon emissions due to its environmental-friendly characteristics. We observed from our findings that there is an inverse relationship between renewable energy consumption and carbon emission, reducing it significantly. This finding is in support of studies from Chen and Mkumbo (2020), Khan et al. (2020) and Inglesi-Lotz and Dogan (2018).

On understanding the impact of FDI on carbon emissions, we observed that FDI at a point could play an insignificant role in carbon emission. However, when it is significant, it is negative. This implies that FDI has a pollution halo effect in the sampled countries. Specifically, our sample countries have stringent environmental regulations that do not permit outmoded production practices and seek cleaner technologies inflow into their countries and green investments. This finding supports the study of Wang et al. (2020a). The significant driver of carbon emissions with the intense effect is economic growth – because the quest to increase output requires energy usage; meanwhile, demand for non-renewable energy far exceeds renewable energy. Therefore, the increase in energy demand in the pursuit of higher output leads to carbon emissions in the long run. However, we observed that carbon emission diminishes when economic growth reaches its optimal level. Specifically, we substantiate the existence of the environmental Kuznets curve (EKC) hypothesis in our sample. This is in agreement with studies from Chen and Mkumbo (2020), Fethi and Rahuma (2019), Kapusuzoğlu (2014), Khan et al. (2020), Mensah et al. (2018) and Vitenu-Sackey (2020).

Notably, we observed that urbanisation proportionally relates to carbon emissions. We contend that the rise in energy demand in urban areas results from migration and human activities increment. This suggests that any further increase in the urban population could increase economic activities that demand energy usage. In contrast, a decrease in urban population growth could reduce energy demand, leading to a reduction in carbon emissions. Some scholarly works agree with our finding – that urbanisation positively leads to carbon emissions significantly (Mensah et al. 2020; Zhang et al. 2017).

After dividing our sample into sub-samples of high-pollution and low-pollution countries, we discovered that the magnitude of the impact of economic policy uncertainties varies; when economic policy uncertainty is high, high-pollution countries appear to have a downward effect on carbon emissions, whereas low-pollution countries appear to have no effect. To put it another way, the economic consequences of policy uncertainty vary significantly in terms of their impact on carbon emissions. R&D, foreign direct investment, urbanisation and renewable energy use all have varying effects on carbon emissions under periods of high economic policy uncertainty, with negative consequences in low-pollution countries but positive benefits in high-pollution countries. According to these findings, the pollution haven hypothesis (PHH) holds true in high-pollution countries. In contrast, the pollution halo effect holds in low-pollution countries, as Ahmad et al. (2021b, a) claim, highlighting the heterogeneous relationship between carbon emissions and economic indicators.

## Conclusion and policy implication

Our study focused on assessing the impact of ecological innovation and economic policy uncertainty on carbon emissions from 2005 to 2018 in a panel study of 18 developed countries. To achieve this objective, we employed second-generation econometric techniques such as CIPS and CADF unit root tests, cross-sectional dependence tests and cointegration tests. We utilised robust techniques for the long-run estimations, including two-stage least square with cross-sectional SUR and panel corrected standard errors (PCSE), generalised method of moment with cross-sectional SUR and panel corrected standard errors and generalised least square with correlation disturbances methods. The preliminary results from the unit root and cointegration tests exhibited stationary and cointegrated data series, while all the variables except two could not substantiate evidence of cross-sectional dependency. In line with this, it required the use of the aforementioned long-run estimators to resolve that issue.

Our empirical analysis concludes that to mitigate carbon emissions, ecological innovation should be increased to avert any economic policy uncertainty that might derail the fortunes of emission mitigation. We can aver that the increasing carbon emissions level requires burgeoning ecological innovations to curtail it – through an increase in research and development and patent application registrations while reducing energy intensity from non-renewable energy sources. Notably, we discovered that the impact of economic policy uncertainty on carbon emissions is diverse. High levels of uncertainty significantly influence carbon emissions only in high-pollution countries but not in low-pollution ones. In the same way, R&D, foreign direct investment, urbanisation and renewable energy usage all have varying effects on carbon emissions. On the other hand, economic growth, patent applications and energy intensity, with a few exceptions, have a symmetric relationship with carbon emissions, regardless of the country’s features. In line with this, we assert that the quest to mitigate carbon emissions should not be a one-size-fits-all approach because not every country’s urbanisation rate, inflows of foreign direct investment, R&D and renewable energy consumption directly affect carbon emissions in the face of economic policy uncertainties.

Considering the findings from our study, we suggest some policy implications: Firstly, the positive relationship between patent, research and development, energy intensity and carbon emissions implies that carbon emissions abatement require numerous ecological innovation. Therefore, research and development should be prioritised towards green technologies and development due to their positive relationship with reducing carbon emissions. This is because the increasing carbon emission level requires more sustainable research and development. Therefore, policymakers should liberate their technological and innovation economies to improve innovation in mitigating carbon emissions. Moreover, energy-saving policies should be promoted to reduce the intensity of non-renewable energy consumption. Secondly, governments should spread development equitably to every place in their countries to curb urban migration to ease the urban infrastructure burden.

Moreover, economic activities should be available in every place, devoid of the level of the place. Even though urbanisation leads to massive development – it also hurts the environment. Increasing demand for economic activities and other basic needs would hamper the environment through greenhouse gas emissions from production and consumption. Finally, we suggest that cleaner technologies be employed in production, and strict environmental regulations should be ensured. Moreover, the cost of renewable energy should be subsidised by governments to increase patronage.

Overall, we confirm the occurrence of aggregation bias and advise that, regardless of a country’s level of development, governments should adjust the stringency of environmental legislation for foreign and host country enterprises based on their environmental performance. It would be a cost-effective approach to get their country on the environmental and economic sustainability pathways. One limitation of our study is the inability to include bootstrapping as our model had more than six covariates; therefore, second-generation cointegration by bootstrapping could not be performed. Future studies can employ second-generation cointegration by bootstrapping techniques.

## Data Availability

Availability of data and materials: The data used in this study can be found in the Mendeley Data repository http://dx.doi.org/10.17632/5p38f8wxg3.1.

## Appendix

Tables 10 and 11

**Table 10:** Variables’ measurement and description

Variables	Measurement	Description	Source
CO_2_	Carbon emission	Carbon dioxide emission per metric tons	OECD database
EPU	Economic policy uncertainty	Index based on newspaper coverage frequency concerning economic-related policy uncertainty, economic forecast uncertainty and tax code expiration	www.policyuncertainty.com
R&D	Research and development	Research and development expenditure (R&D) US$ million	OECD database
EINT	Energy intensity	Primary energy use/GDP per capita US$ million	World Development Indicators, World Bank
PT	Technological innovation	Patent registration (number of registration)	
GDP_CAP	Economic growth	GDP per capita US$ thousand PPP	OECD database
GDP_CAP2	Quadratic term of economic growth	Squared of natural logarithm of GDP_CAP	Authors’ calculation
URP	Urbanisation	Urban population (people living in metropolitan and urban cities as % of the total population)	World Development Indicators-World Bank
FDI	Foreign direct investment	Net inflows US$ million	OECD database
RE	Renewable Energy Consumption	% of total final energy consumption	OECD database

**Table 11:** Classification of countries included in the estimations

**Sampled countries**	
Australia (high polluter)	China (high polluter)
Canada (high polluter)	Russia (high polluter)
Korea (low polluter)	Mexico (low polluter)
Greece (low polluter)	United Kingdom (high polluter)
The Netherlands (low polluter)	USA (high polluter)
France (high polluter)	Chile (low polluter)
Germany (high polluter)	Ireland (low polluter)
Spain (low polluter)	Italy (low polluter)
Sweden (low polluter)	Japan (high polluter)

## Footnotes

1 This adapts the works of Mensah et al. (2020), Chen and Mkumbo (2020), Vitenu-Sackey (2020) and Fethi and Rahuma (2019).

2 See https://datacatalog.worldbank.org/search/dataset/0037712

3 The rankings of the countries can be found at https://www.carbonbrief.org/analysis-which-countries-are-historically-responsible-for-climate-change.
